# Sepsis as a Pan-Endocrine Illness—Endocrine Disorders in Septic Patients

**DOI:** 10.3390/jcm10102075

**Published:** 2021-05-12

**Authors:** Weronika Wasyluk, Martyna Wasyluk, Agnieszka Zwolak

**Affiliations:** 1 Chair of Internal Medicine and Department of Internal Medicine in Nursing, Faculty of Health Sciences, Medical University of Lublin, 20-093 Lublin, Poland; agnieszka.zwolak@umlub.pl; 2 Doctoral School, Medical University of Lublin, 20-093 Lublin, Poland; 3 Student’s Scientific Association at Chair of Internal Medicine and Department of Internal Medicine in Nursing, Faculty of Health Sciences, Medical University of Lublin, 20-093 Lublin, Poland; martynaxwasyluk@gmail.com

**Keywords:** sepsis, septic shock, inflammation, critical illness, intensive care, metabolism, hormones, endocrinology

## Abstract

Sepsis is defined as “life-threatening organ dysfunction caused by a dysregulated host response to infection”. One of the elements of dysregulated host response is an endocrine system disorder. Changes in its functioning in the course of sepsis affect almost all hormonal axes. In sepsis, a function disturbance of the hypothalamic–pituitary–adrenal axis has been described, in the range of which the most important seems to be hypercortisolemia in the acute phase. Imbalance in the hypothalamic–pituitary–thyroid axis is also described. The most typical manifestation is a triiodothyronine concentration decrease and reverse triiodothyronine concentration increase. In the somatotropic axis, a change in the secretion pattern of growth hormone and peripheral resistance to this hormone has been described. In the hypothalamic–pituitary–gonadal axis, the reduction in testosterone concentration in men and the stress-induced “hypothalamic amenorrhea” in women have been described. Catecholamine and β-adrenergic stimulation disorders have also been reported. Disorders in the endocrine system are part of the “dysregulated host response to infection”. They may also affect other components of this dysregulated response, such as metabolism. Hormonal changes occurring in the course of sepsis require further research, not only in order to explore their potential significance in therapy, but also due to their promising prognostic value.

## 1. Introduction

According to the latest guidelines, sepsis is defined as “life-threatening organ dysfunction caused by a dysregulated host response to infection” [1]. Contrary to the older definitions, the current one not only focuses on the inflammation, but also indicates systemic disturbance of homeostasis. Dysregulated response is highly evident in the endocrine system functioning. A full understanding of the role of hormonal changes in the pathophysiology of sepsis will allow both clinicians to better manage currently available interventions, such as the use of exogenous hormones and nutrition treatment, and scientists to find grip points for potential new therapies. Therefore, the aim of this review is to analyze the current knowledge of changes in the endocrine system in the course of sepsis and their effect on metabolism.

## 2. The Pathogenesis of Sepsis

**Figure 1:**
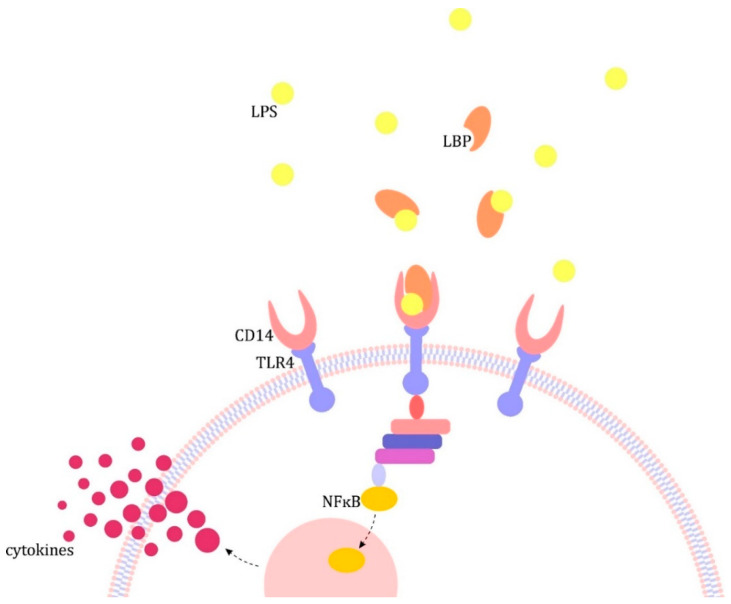
Pathogenesis of sepsis with the example of lipopolysaccharide (LPS). The major patho-mechanism is the formation of complexes in the blood with lipopolysaccharide binding protein (LBP), which in turn binds to CD14 receptors present on monocytes/macrophages and neutrophils and circulating in the plasma. The resulting complex activates Toll-like receptors (TLRs), which transmit the signal inside the cell, leading to the translocation of the nuclear factor kB (NF-kB) to the cell nucleus and activation of pro-inflammatory cytokine gene promoters, including interleukin (IL) 1 and tumor necrosis factor α (TNF-α) [2].

The inflammation response to infection in sepsis develops as a result of the body’s reaction to microbial components, including lipopolysaccharide (endotoxin, LPS) found in gram-negative bacteria, lipoteichoic acid found in gram-positive bacteria and other fragments of microorganisms. They are bound by cell receptors of the immune system and endothelium, which initiate the cascade of reactions leading to the release of pro-inflammatory cytokines. Figure 1 shows this using the LPS as an example [2].

Pro-inflammatory cytokines contribute to the procoagulant state, increased production of free oxygen radicals (ROS) and nitric oxide (NO). Excessive production of NO is considered to be one of the causes of vasodilation and impaired response to vasoconstrictive factors (vascular hypo-reactivity), which is a component of the pathophysiology of septic shock. [3,4] Compensatory to the pro-inflammatory response, the activation of anti-inflammatory mediators (including IL-4, IL-10, IL-13) takes place. After varying time, the pathways for the synthesis of pro-inflammatory mediators may be exhausted and the immune balance may shift towards immunosuppression. This condition is characterized by increased levels of anti-inflammatory cytokines, T-cell anergy, increased apoptosis and impaired immune cell function [5,6]. In addition to the uncontrolled inflammatory response, sepsis is also characterized by dysregulated hemostasis—the inflammatory process activates the coagulation system, which can lead to thrombosis in the microcirculation, which in turn can cause organ hypoxia. Moreover, sepsis disrupts the integrity and function of the endothelium—LPS, cytokines, ROS and other mediators released in the course of sepsis cause damage to the shedding of the glycocalyx, leading to a drastic increase in vascular permeability. This results in the displacement of fluids from the intravascular to the extravascular space, which causes tissue oedema. Sepsis also increases the release of adhesion molecules from the endothelium, which favors the accumulation of neutrophils, that may lead to tissue damage [7].

## 3. Endocrine Disorders in the Course of Sepsis

**Figure 2:**
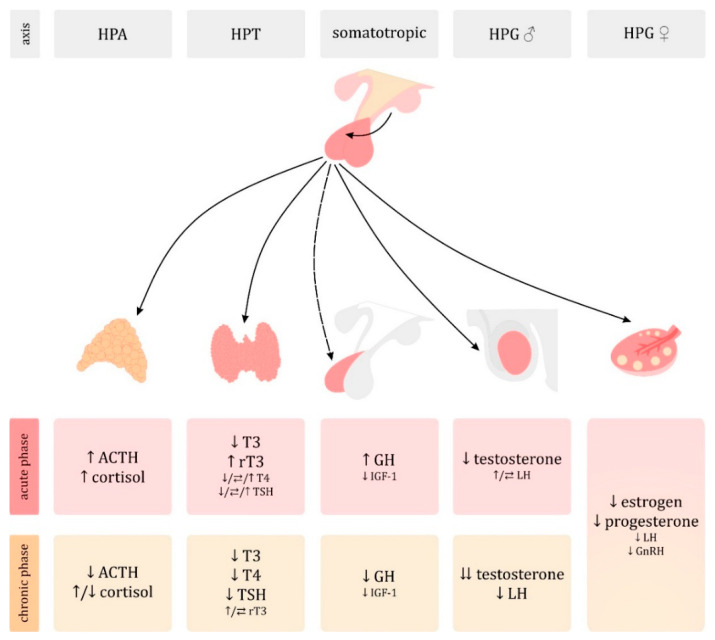
Hormonal changes in sepsis—differences between acute and chronic phases. Symbols: ↑ − increase; ↓ − decrease, ⇆ − normal, ♂ − male, ♀ − female. Abbreviations: ACTH, adrenocorticotropic hormone (corticotropin); GH, growth hormone (somatropin); GnRH, gonadotropin-releasing hormone (gonado-liberin); HPA, hypothalamic-pituitary-adrenal; HPG, hypothalamic–pituitary–gonadal; HPT, hypothalamic–pituitary–thyroid; IGF-1, insulin-like growth factor I; LH, luteinizing hormone; rT3, reverse triiodothyronine; T3, triiodothyronine; T4, thyroxine; TSH, thyrotropin.

A wide variety of endocrine disorders in sepsis have been described. The concept of the constancy of the internal environment of organism, also known as milieu intérieur, was introduced in 1865 by the French physiologist Claude Bernard [8]. 60 years later the physiologist Walter Bradford Cannon introduced the concept of homeostasis [9] based on its assumptions. Cannon also presented the theory according to which, in the case of the threat to the maintenance of homeostasis, the sympathetic nervous system and the adrenal medulla are activated. He believed that these effectors cooperate to restore homeostasis, working as one unit of the sympathoadrenal system [10,11]. It is now known that the body’s response to highly stressful stimuli, such as sepsis, is a complex composition of interactions between the autonomic nervous system, the immune system and the endocrine system. As in the historical sources described, it is assumed that this reaction is aimed at ensuring survival despite unfavorable conditions. In sepsis, the threat is recognized by the immune system, which can then be considered a sensory organ. It signals the presence of pathogens to the central nervous system (CNS) via various pathways, including the release of cytokines and neurotoxic mediators, signaling through the vagus nerve and circumventricular organs (neurohypophysis) [12]. These pathways transmit stimuli afferently to the CNS, triggering a response from the autonomic nervous system and neurohormonal axes—the hypothalamic–pituitary–adrenal axis (HPA), the hypothalamic–pituitary–thyroid axis (HPT), the somatotropic axis and the hypothalamic–pituitary–gonadal (HPG). It should also be emphasized that there is a difference between endocrine and metabolic changes in the initial acute and later chronic stages of sepsis, and that the time frames of these phases are highly variable in individual patients (Figure 2). Catecholamines and insulin resistance are other areas of sepsis-related hormonal changes that affect metabolism. A detailed discussion of these hormonal disorders is provided below.

### 3.1. Hypothalamic–Pituitary–Adrenal (HPA) Axis

Under physiological conditions, the hypothalamus produces pulsatile cortico-liberin (CRH), which stimulates the pituitary gland to pulsate the secretion of adrenocorticotropic hormone (corticotropin, ACTH), which in turn regulates the production of cortisol and dehydroepiandrosterone (DHEA) by the adrenal cortex. It also has a minor effect on the secretion of aldosterone (which is regulated mainly by the renin–angiotensin system). Cortisol, by a negative feedback mechanism, inhibits the secretion of CRH and ACTH.

**Figure 3:**
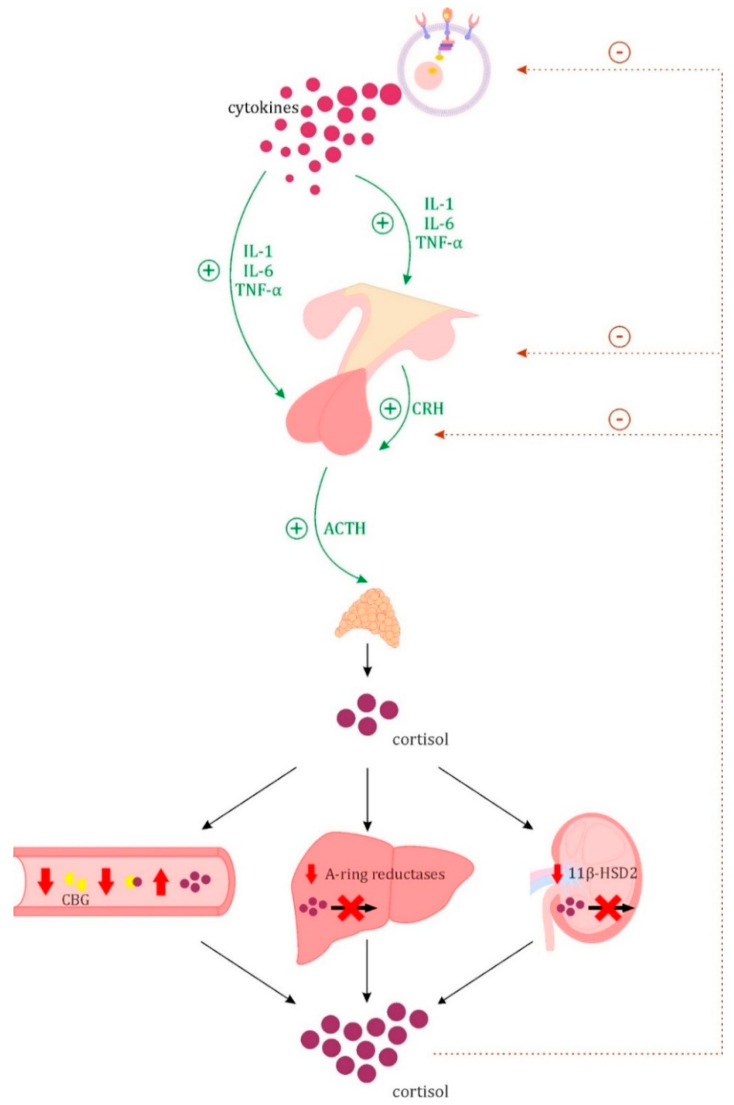
Changes in the action of the hypothalamic–pituitary–adrenal axis (HPA) in sepsis. Inflammatory cytokines (IL-1, IL-6, TNF-α) increase the secretion of CRH in the hypothalamus and ACTH in the anterior pituitary, which increases the secretion of cortisol by the adrenal glands. In the course of sepsis, there is also impaired cortisol metabolism in the liver by A-ring reductases, and in the kidneys, by 11β-hydroxysteroid dehydrogenase type 2 (11β-HSD2). Another mechanism responsible for maintaining high blood cortisol levels may be the decreased level of corticosteroid-binding globulin (CBG). The described disorders lead to hypercortisolemia. High levels of cortisol, through a feedback mechanism, can inhibit the secretion of the HPA axis hormones by the hypothalamus and pituitary gland.

Activating the HPA axis is one of the primary responses not only to sepsis, but also to acute stress in general, and is essential for survival. In critically ill patients the blood cortisol concentration increases and its circadian rhythm of secretion is lost [13]. The influence of many cytokines on the secretory activity of the HPA axis has been demonstrated, but its main immune stimulators are IL-1, IL-6 and TNF-α, which increase the secretion of CRH and ACTH (Figure 3) [14,15]. The hormonal response of the hypothalamus and pituitary gland to cytokines is biphasic and is characterized by an increase in ACTH concentration in the acute phase, turning into a decrease in the chronic phase [15].

It is also possible that cytokines have a direct effect on the adrenal glands, regulating the secretion of cortisol [16,17,18].

In the literature from the early 1990s, the term immune–hypothalamic–pituitary–adrenal axis [19] appears, which can be considered accurate. Cortisol has an immunosuppressive effect, reducing the concentration of interleukins, so the regulation of the entire system is based on feedback, which is typical for the hormonal axes. Among the stimulators of cortisol secretion, vasopressin, endothelin, substance P and atrial natriuretic factors are also mentioned [20].

In addition to the increased production of cortisol by the adrenal glands, impaired glucocorticoid (GCs) metabolism also contributes to its increase in concentration in the blood in critically ill patients [21]. Cortisol is metabolized mainly in the liver (A-ring reductases) and in the kidneys (11β-hydroxysteroid dehydrogenase type 2). A significant increase in the half-life of cortisol during sepsis has been reported [21,22]. It has been shown that in critically ill patients there is an increase in circulating bile acids, which may inhibit cortisol metabolizing enzymes [23,24,25,26,27]. Another mechanism, potentially responsible for maintaining high blood cortisol levels, is decreased level of corticosteroid-binding globulin (transcortins, CBG). The reduction of CBG concentration occurs in patients during the acute phase reaction, due to the mechanism of increased protein degradation by elastase. This results in greater availability of free cortisol [28,29,30,31]. Additionally to the above-mentioned mechanisms causing a change in blood cortisol concentration, the biological effect of its action may also be influenced by changes in the expression of glucocorticoid receptors (GR). Both increases and decreases in receptor expression have been reported in sepsis patients. Expression reduction is more often associated with a more severe course and worse prognosis. It has been described that the reduction in the number and the weakening of the function of GR receptors is associated with a significantly increased amount of cytokines [32,33,34,35,36,37,38]. This phenomenon may lead to resistance to GCs [39].

Regardless of details of the mechanism, sepsis is characterized by hypercortisolemia, which is associated with adaptive benefits and deleterious complications. Benefits include inhibition of inflammation and maintenance of vascular tone, catecholamine sensitivity and endothelial integrity. Complications consist of increased susceptibility to infections and myopathy. Cortisol affects metabolism. It has, among others, hyperglycemic effect. As an adaptation to the increased energy cell needs, this occurs as beneficial; however, long-term hyperglycemia may have negative consequences [40].

However, sometimes, the typical HPA axis response is disturbed. This condition is termed critical illness-related corticosteroid insufficiency (CIRCI) [15]. This name was proposed in 2008 by an international task force by the American College of Critical Care Medicine (ACCM) as a term for dysfunction of the HPA axis in critical illness (including sepsis). They defined CIRCI as “inadequate cellular corticosteroid activity for the severity of the patient’s illness” and emphasized that in this unit adrenal insufficiency may result from dysfunction anywhere on the HPA axis. Usually the cause is the action of inflammatory mediators, but failure may also originate from structural damage. According to the recommendations, terms such as absolute or relative adrenal insufficiency should be avoided when referring to critically ill patients [41]. For this review, it is important to note that CIRCI may affect the anti-inflammatory and pro-inflammatory processes balance—thus influencing immune and metabolic disorders [41].

In 2017, the joint guidelines of the Society of Critical Care Medicine (SCCM) and the European Society of Intensive Care Medicine (ESICM) were published to update the 2008 consensus on CIRCI. This document states that “CIRCI is characterized by dysregulated systemic inflammation resulting from inadequate intracellular glucocorticoid-mediated anti-inflammatory activity for the severity of the patient’s critical illness” [42]. Attention was also paid to the growing amount of evidence for the presence of CIRCI in a wide spectrum of critical diseases, and the relationship between the occurrence of this entity and length of ICU stay and mortality. In light of this information, the importance of critical care providers’ understanding of the pathogenesis and treatment of CIRCI was emphasized [42]. These issues were discussed in detail in the publications of Annane et al. [15,42].

The mechanisms involved in the pathogenesis of CIRCI can be divided into four main groups—endocrine cell damage, dysregulation of the HPA axis, altered cortisol metabolism and tissue resistance to GCs [15]. Ischemic or hemorrhagic damage of the elements of the HPA axis happens relatively rarely during sepsis. The syndrome caused by both adrenal glands hemorrhage was described over 100 years ago and called Waterhause-Fridrichsen syndrome, after the names of the doctors who first defined it [43,44]. Ischemic and hemorrhagic changes in the course of sepsis have also been reported in the hypothalamus and pituitary gland [45]. Steroidogenesis disorders may also occur without structural damage to the adrenal glands. The dysregulation of the HPA axis results from the aforementioned biphasic hormonal response of the pituitary and hypothalamus to the action of cytokines. This leads to a decreased concentration of ACTH in the chronic phase of the critical illness [15]. ACTH secretion may be feedback suppressed by the concentration of cortisol, which remains high in a mechanism independent of the HPA axis (Figure 3) [15]. It has also been suggested that NO may mediate the reduction in ACTH synthesis following sepsis [46,47]. The inhibition of ACTH synthesis may have an iatrogenic basis, associated with the therapeutic use of GCs, but also with the administration of drugs such as azole antifungals, opioid analgesics and psychoactive drugs [48]. Atrophy of the adrenal cortex, caused by prolonged insufficient ACTH stimulation, has also been described [49]. Another group of mechanisms involves the processes leading to altered cortisol metabolism. In 2010, Polito et al. proposed cholesteryl ester deficiency which results in adrenal lipid store depletion as a potential mechanism of adrenal insufficiency in the course of sepsis. In the study involving histological analysis of the adrenal cortex in deceased patients and in the animal model of sepsis, diffuse lipid depletion in zona fasciculata was shown [50]. Moreover, also in this group of mechanisms, the influence of iatrogenic factors is possible—steroidogenesis can be inhibited by drugs at the stage of several enzymatic reactions [48]. The last mechanism, GC resistance, was discussed earlier.

### 3.2. Hypothalamic–Pituitary–Thyroid (HPT) Axis

The thyroid hormones, thyroxine (T4) and triiodothyronine (T3), play a key role in the differentiation, growth and metabolism of all tissues. Physiologically, their concentration in the blood is regulated by the hypothalamus and pituitary gland, secreting thyroliberin (TRH) and thyrotropin (TSH) respectively, and controlled in a negative feedback mechanism by thyroid hormones present in the blood. The thyroid gland mainly produces a less active form of the hormone T4, which is converted to the more active T3 or inactive reverse triiodothyronine (rT3) in peripheral tissues by deiodinases. In critically ill patients there are significant disturbances in the HPT axis [51].

The initial response of patients to critical illness, as sepsis and septic shock, includes a sudden fall in T3 and an increase in rT3. Reports on TSH and T4 levels are more divergent—increase and decrease in concentration, as well as lack of significant changes, are reported [52,53,54,55,56,57]. Changes in blood levels of HPT axis hormones, most often including low T3 levels, normal TSH levels and increased rT3 levels, have been termed nonthyroidal illness syndrome (NTIS), formerly also known as euthyroid sick syndrome or low T3 syndrome [58]. The theories explaining the role of this phenomenon include the compensatory role in relation to the oxidative stress associated with acute disease [59,60] and the adaptive role—as an attempt to reduce energy consumption and protect against protein catabolism [61,62,63]. A number of changes accompanying NTIS, which may be responsible for disturbances in hormone levels, such as the activity of deiodinases, the concentration of thyroid hormone binding proteins, the activity of the nuclear thyroid hormone receptor, the inhibition of hormone binding, transport and metabolism by free fatty acids and bilirubin, have been described [58,64,65]. There have also been suggestions that this phenomenon is related to the presence of cytokines, but the efficacy of cytokine antagonists in reversing NTIS has not been demonstrated [66].

Compared to the acute phase, mainly characterized by changes in the peripheral hormone metabolism, the chronic phase is characterized by both peripheral changes and central adaptation. After a few days of illness, critically ill patients still exhibit a reduced level of T3, accompanied by T4 decrease, and a decrease in pituitary gland pulsatile TSH secretion, correlating with decreased expression of genes for TRH in the hypothalamus [67,68,69,70]. Reports on the possibility of restoring normal TSH, T3 and T4 levels by TRH infusion (especially in combination with growth hormone secretagogues) support the central character of changes in the chronic critical phase of the disease [71].

The causes of disturbances in the central regulation of the HPT axis remain unclear, with cytokines, neuropeptide Y, hypercortisolemia, changes in the activity of hypothalamic deiodinase and thyroid hormone transporter expression among the potential causes. Iatrogenic causes, including treatment of patients with dopamine and GCs, are also taken into account [20].

The prognostic significance of changes in the HPT axis hormones levels has also been shown. Negative correlation between mortality and the levels of T3 and T4 has been described in sepsis patients [54,72,73]. There is a positive and negative correlation between TSH levels and mortality [72,74]. Moreover, one study showed that the Acute Physiology and Chronic Health Evaluation II (APACHE II) score was negatively correlated with fT3 and fT4 and positively with TSH [75].

### 3.3. Somatotropic Axis

Growth hormone (somatropin, GH) is pulsatively secreted by the anterior pituitary gland. Physiologically, GH secretion is controlled by the hypothalamus through stimulating growth hormone releasing hormones (somatoliberin, GHRH) and inhibitory somatostatin (SRIF) [76]. Moreover, the enteric hormone ghrelin is also involved in the regulation of GH secretion [77,78,79]. This corresponds to physiological stimuli, such as sleep and wakefulness, food intake, physical activity and stress [76]. GH has direct and indirect effects. Its indirect action is mediated by somatomedins, including insulin-like growth factor I (IGF-I). In response to acute stress, the pattern of GH secretion changes—the frequency, amplitude and level of intra-pulsation increase [20]. Peripheral GH resistance, characterized by decreased IGF-1 secretion, has also been described [80,81,82,83,84].

It is assumed that these changes may be adaptive—GH acts in a lipolytic and hyperglycemic manner, which increases the availability of glucose and fatty acids; at the same time the anabolic effect of GH mediated by IGF-I and suppression of GH secretion by IGF-I through a negative feedback mechanism are suppressed [85,86].

In addition to an increase in GH concentration and a decrease in IGF-I concentration in the course of sepsis, a decrease in the concentration of insulin-like growth factor-binding protein 3 (IGFBP-3) and the increase in IGFBP-1, IGFBP-2, IGFBP-4 are also described [82,87,88].

As in the previously described axes, correlation between the concentration of hormones, the patient’s condition and treatment outcome has been demonstrated. GH levels have been shown to be higher in patients with septic shock compared to patients with sepsis and also in patients who did not survive compared to those who survived. IGF-I concentration is higher in sepsis patients compared to septic shock patients. This also negatively correlates with sepsis severity and mortality [89,90,91].

In the chronic phase, pulsatile GH secretion is reduced and IGF-I and IGFPBP-3 levels are low. This condition may hinder tissue reconstruction and contribute to the so-called wasting syndrome [92]. Evidence has also been presented of the antioxidant and anti-inflammatory effects of IGF-1. It is assumed that its low levels may exacerbate oxidative stress injury in sepsis patients [93,94,95]

Another hormone associated with the somatotropic axis is ghrelin. This is a relatively recently discovered hormone that acts on the GH secretagogues receptor (GHS-R) and stimulates the secretion of GH by the pituitary gland [96]. Ghrelin, mainly secreted by stomach fundus cells, acts as an appetite stimulant, but has many other effects. It is believed to play an important role in the regulation of metabolism [77]. The increase in ghrelin concentration in sepsis has been observed in patients and in the experimental model [97,98]. Increased concentration has been reported to positively correlate with the levels of TNF-α, IL-6 and C reactive protein (CRP) [98]. It may be beneficial in patients with sepsis due to its anti-inflammatory effect through the modulation of cytokine secretion [99,100]. Its beneficial effect is also described in the context of reducing catabolism, improving hemodynamic, weakening immunosuppression and enhancing autophagic repair processes in experimental sepsis models [97,101,102].

### 3.4. Hypothalamic–Pituitary–Gonadal (HPG) Axis

The HPG axis includes the hypothalamus, pulsating secreting gonadotropin-releasing hormone (gonado-liberin, GnRH), inducing the anterior pituitary gland to secrete gonadotrophins: luteinizing hormone (LH) and follicle stimulating hormone (FSH) [103]. In women, LH stimulates the ovary to produce androgens, while FSH contributes to their aromatization to estrogens. In men, LH stimulates the Leydig cells in the testes to produce androgens, while FSH, together with testosterone, stimulates Sertoli cells to spermatogenesis. Moreover, sex steroids affect, in a negative feedback mechanism, the hypothalamus and pituitary gland, inhibiting the secretion of GnRH and gonadotrophins [103]. Other hormones involved in the regulation of the HPG axis include prolactin (PRL), secreted in pulses by the anterior pituitary gland. Under physiological conditions, the control of its secretion depends mainly on dopamine flow from the hypothalamus to the anterior pituitary gland via the hypophyseal portal system [104]. It has also been shown that PRL has immunomodulatory functions [105].

In men, a critical illness causes a decrease in testosterone levels in the first place (despite increasing or not changing LH levels). This is due to the direct effect of cytokines on Leydig cells and the increased peripheral aromatization of androgens [106,107,108,109,110]. It has been reported that during septic shock, increased aromatization of androgens causes an increase in 17β-estradiol (E2) concentration and a decrease in testosterone concentration [111]. In the chronic phase of critical illness, persistent androgen aromatization and primary hypogonadism in men have been described—testosterone concentration is extremely low, with low pulsatile LH secretion and mean LH concentrations [110,112,113,114,115]. The supply of exogenous GnRH shows limited effectiveness in restoring the normal function of the HPG axis, which suggests the coexistence of central and peripheral disorders [115]. Since testosterone is a strong anabolic hormone, lowering its concentration may be a form of adaptation to the increased energy needs of the body, but long-term low levels may contribute to the development of catabolic wasting syndrome in a patient. A negative correlation has been demonstrated between testosterone concentration and mortality in critically ill men [107].

In women, stress-induced hypothalamic amenorrhea has been described as a consequence of critical illness. The pathogenesis is based on the bidirectional cross-talk between the HPA axis (especially CRH) and the HPG axis [116]. Stress promotes both eutopic (in the hypothalamus) and ectopic (in immune cells and the reproductive system) secretion of corticotropin releasing factor (CRF), which inhibits hypothalamic GnRH secretion, while GCs inhibit pituitary LH secretion and the production of estrogen and progesterone by the ovaries [117,118,119].

In the course of sepsis, the secretion of the PRL changes. An increase in its concentration in the acute phase of sepsis and the correlation between its concentration and the results of the Simplified Acute Physiology Score III (SAPS III) and APACHE II has been demonstrated [120]. In the chronic phase of critical illness, the secretion of PRL is suppressed [68].

Interestingly, sexual dimorphism in morbidity and mortality from sepsis has been described. There are many studies showing higher incidence [121,122,123] and mortality [123,124,125] in men (although there are also studies that do not confirm these relationships [122,126] or present them only for women up to 40 years of age [127]). Animal studies have shown sexual dimorphism in the immune response to sepsis. Described are: the role of female gonadal hormones and their receptors in compensating for cardiovascular disorders induced by endotoxemia in rats [128], beneficial effects of estradiol on phagocytic and bactericidal function of immune cells [129], and estrogen-driven, innate antibody-mediated immunological strategy conferred protection providing protection against Escherichia coli in female rats [130]. This sexual dimorphism may be due to the pro-inflammatory effect of estradiol in women and the suppressive effect of testosterone on the immune system in men [131].

### 3.5. Catecholamines, β-Adrenergic Stimulation and Insulin Resistance

In the course of sepsis, the adrenergic nervous system is activated. This causes the release of catecholamines from the adrenal medulla. They affect, inter alia, the metabolism, increasing catabolism [132]. Activation of the adrenergic system also mediates insulin resistance in patients with sepsis [133]. Adrenaline and β-adrenergic stimulation has been shown to inhibit insulin-dependent glucose uptake in skeletal muscle [134]. β-adrenergic stimulation may also inhibit the insulin-signaling pathway. Inhibition of insulin signaling has been described for the insulin receptor, glucose transporter (GLUT) and the glycolysis pathway [135,136]. Moreover, activation of the adrenergic system has a hyperglycemic effect—catecholamines increase hepatic glycogenolysis and gluconeogenesis [137]. In addition, β-adrenergic stimulation activates lipolysis, that leads to a reduction in glucose oxidation (Randle cycle) [138], which may also exacerbate hyperglycemia.

## 4. Conclusions

In the course of sepsis, there are changes in almost all functioning hormonal axes. It is suggested that some of these changes are adaptive, but there is no doubt that all of them may have an impact on the patient’s clinical condition. It is also worth bearing in mind that a patient’s endocrine profile is not the same for the duration of the disease, but changes over time. The differences are especially visible between the acute and the chronic phase. Disorders in the endocrine system are part of the “dysregulated host response to infection” in the definition of sepsis and may affect other components of this dysregulated response, such as metabolism. Hormonal changes, occurring in the course of sepsis, require further research not only in order to explore their potential significance in therapy, but also due to their promising prognostic value.

## Data Availability

Not applicable.

## References

[1] Singer M., Deutschman C.S., Seymour C.W., Shankar-Hari M., Annane D., Bauer M., Bellomo R., Bernard G.R., Chiche J.D., Coopersmith C.M. (2016). The third international consensus definitions for sepsis and septic shock (sepsis-3). JAMA.

[2] Cohen J. (2002). The immunopathogenesis of sepsis. Nature.

[3] Carré J., Singer M., Moncada S. (2007). Nitric Oxide. Mechanisms of Sepsis-Induced Organ Dysfunction and Recovery.

[4] Singer M. (2007). Dysfunction of the Bioenergetic Pathway.

[5] Hotchkiss R.S., Karl I.E. (2003). The pathophysiology and treatment of sepsis. N. Engl. J. Med..

[6] Carré J.E., Singer M. (2008). Cellular energetic metabolism in sepsis: The need for a systems approach. Biochim. Et Biophys. Acta Bioenergy.

[7] Kübler A., Kübler A. (2017). Patogeneza. Sepsa.

[8] Bernard C. (1957). An Introduction to the Study of Experimental Medicine.

[9] Cannon W.B., Pettit A. (1926). Physiological regulation of normal states: Some tentative postulates concerning biological homeostatics. A Charles Richet: Ses Amis, Ses Collègues, Ses Élèves.

[10] Cannon W.B. (1915). Bodily Changes in Pain, Hunger, Fear and Rage, An Account of Recent Researches into the Function of Emotional Excitement.

[11] Goldstein D.S., Kopin I.J. (2007). Evolution of concepts of stress. Stress.

[12] Gheorghiţă V., Barbu A.E., Gheorghiu M.L., Căruntu F.A. (2015). Endocrine dysfunction in sepsis: A beneficial or deleterious host response?. GERMS.

[13] Cooper M.S., Stewart P.M. (2003). Corticosteroid insufficiency in acutely ill patients. N. Engl. J. Med..

[14] Turnbull A.V., Rivier C.L. (1999). Regulation of the hypothalamic-pituitary-adrenal axis by cytokines: Actions and mechanisms of action. Physiol. Rev..

[15] Annane D., Pastores S.M., Arlt W., Balk R.A., Beishuizen A., Briegel J., Carcillo J., Christ-Crain M., Cooper M.S., Marik P.E. (2017). Critical illness-related corticosteroid insufficiency (CIRCI): A narrative review from a Multispecialty Task Force of the Society of Critical Care Medicine (SCCM) and the European Society of Intensive Care Medicine (ESICM). Intensive Care Med..

[16] Kanczkowski W., Sue M., Zacharowski K., Reincke M., Bornstein S.R. (2015). The role of adrenal gland microenvironment in the HPA axis function and dysfunction during sepsis. Mol. Cell. Endocrinol..

[17] Mikhaylova I.V., Kuulasmaa T., Jääskeläinen J., Voutilainen R. (2007). Tumor necrosis factor-α regulates steroidogenesis, apoptosis, and cell viability in the human adrenocortical cell line NCI-H295R. Endocrinology.

[18] Engström L., Rosén K., Angel A., Fyrberg A., Mackerlova L., Konsman J.P., Engblom D., Blomqvist A. (2008). Systemic immune challenge activates an intrinsically regulated local inflammatory circuit in the adrenal gland. Endocrinology.

[19] Bateman A., Singh A., Kral T., Solomon S. (1989). The immune-hypothalamic-pituitary-adrenal axis. Endocr. Rev..

[20] Ingels C., Gunst J., van den Berghe G. (2018). Endocrine and Metabolic Alterations in Sepsis and Implications for Treatment. Crit. Care Clin..

[21] Boonen E., Vervenne H., Meersseman P., Andrew R., Mortier L., Declercq P.E., Vanwijngaerden Y.M., Spriet I., Wouters P.J., Vander Perre S. (2013). Reduced Cortisol Metabolism during Critical Illness. N. Engl. J. Med..

[22] Boonen E., Meersseman P., Vervenne H., Meyfroidt G., Guïza F., Wouters P.J., Veldhuis J.D., Van den Berghe G. (2014). Reduced nocturnal ACTH-driven cortisol secretion during critical illness. Am. J. Physiol. Endocrinol. Metab..

[23] Ackermann D., Vogt B., Escher G., Dick B., Reichen J., Frey B.M., Frey F.J. (1999). Inhibition of 11β-hydroxysteroid dehydrogenase by bile acids in rats with cirrhosis. Hepatology.

[24] McNeilly A.D., Macfarlane D.P., O’Flaherty E., Livingstone D.E., Mitić T., McConnell K.M., McKenzie S.M., Davies E., Reynolds R.M., Thiesson H.C. (2010). Bile acids modulate glucocorticoid metabolism and the hypothalamic-pituitary-adrenal axis in obstructive jaundice. J. Hepatol..

[25] Stauffer A.T., Rochat M.K., Dick B., Frey F.J. (2002). Odermatt, Chenodeoxycholic acid and deoxycholic acid inhibit 11β-hydroxysteroid dehydrogenase type 2 and cause cortisol-induced transcriptional activation of the mineralocorticoid receptor. J. Biol. Chem..

[26] Vanwijngaerden Y.M., Wauters J., Langouche L., Vander Perre S., Liddle C., Coulter S., Vanderborght S., Roskams T., Wilmer A., Van den Berghe G. (2011). Critical illness evokes elevated circulating bile acids related to altered hepatic transporter and nuclear receptor expression. Hepatology.

[27] Thomas H. (2017). Sepsis: Bile acids promote inflammation in cholestasis-associated sepsis. Nat. Rev. Gastroenterol. Hepatol..

[28] Pugeat M., Bonneton A., Perrot D., Rocle-Nicolas B., Lejeune H., Grenot C., Dechaud H., Brebant C., Motin J., Cuilleron C.Y. (1989). Decreased immunoreactivity and binding activity of corticosteroid-binding globulin in serum in septic shock. Clin. Chem..

[29] Bae Y.J., Kratzsch J. (2015). Corticosteroid-binding globulin: Modulating mechanisms of bioavailability of cortisol and its clinical implications. Best Pract. Res. Clin. Endocrinol. Metab..

[30] Nenke M.A., Rankin W., Chapman M.J., Stevens N.E., Diener K.R., Hayball J.D., Lewis J.G., Torpy D.J. (2015). Depletion of high-affinity corticosteroid-binding globulin corresponds to illness severity in sepsis and septic shock; Clinical implications. Clin. Endocrinol..

[31] Ho J.T., Al-Musalhi H., Chapman M.J., Quach T., Thomas P.D., Bagley C.J., Lewis J.G., Torpy D.J. (2006). Septic shock and sepsis: A comparison of total and free plasma cortisol levels. J. Clin. Endocrinol. Metab..

[32] Molijn G.J., Spek J.J., Van Uffelen J.C., De Jong F.H., Brinkmann A.O., Bruining H.A., Lamberts S.W., Koper J.W. (1995). Differential adaptation of glucocorticoid sensitivity of peripheral blood mononuclear leukocytes in patients with sepsis or septic shock. J. Clin. Endocrinol. Metab..

[33] Cohen J., Pretorius C.J., Ungerer J.P., Cardinal J., Blumenthal A., Presneill J., Gatica-Andrades M., Jarrett P., Lassig-Smith M., Stuart J. (2016). Glucocorticoid sensitivity is highly variable in critically ill patients with septic shock and is associated with disease severity. Crit. Care Med..

[34] Alder M.N., Opoka A.M., Wong H.R. (2018). The glucocorticoid receptor and cortisol levels in pediatric septic shock. Crit. Care.

[35] Jenniskens M., Weckx R., Dufour T., Vander Perre S., Pauwels L., Derde S., Téblick A., Güiza F., Van den Berghe G., Langouche L. (2018). The hepatic glucocorticoid receptor is crucial for cortisol homeostasis and sepsis survival in humans and Male mice. Endocrinology.

[36] Abraham M.N., Jimenez D.M., Fernandes T.D., Deutschman C.S. (2018). Cecal Ligation and Puncture Alters Glucocorticoid Receptor Expression. Crit. Care Med..

[37] Ganesh K., Sharma R., Varghese J., Pillai M.G.K. (2016). A profile of metabolic acidosis in patients with sepsis in an Intensive Care Unit setting. Int. J. Crit. Illn. Inj. Sci..

[38] Vardas K., Ilia S., Sertedaki A., Charmandari E., Briassouli E., Goukos D., Apostolou K., Psarra K., Botoula E., Tsagarakis S. (2017). Increased glucocorticoid receptor expression in sepsis is related to heat shock proteins, cytokines, and cortisol and is associated with increased mortality. Intensive Care Med. Exp..

[39] Dendoncker K., Libert C. (2017). Glucocorticoid resistance as a major drive in sepsis pathology. Cytokine Growth Factor Rev..

[40] Arafah B.M. (2006). Review: Hypothalamic pituitary adrenal function during critical illness: Limitations of current assessment methods. J. Clin. Endocrinol. Metab..

[41] Marik P.E., Pastores S.M., Annane D., Meduri G.U., Sprung C.L., Arlt W., Keh D., Briegel J., Beishuizen A., Dimopoulou I. (2008). Recommendations for the diagnosis and management of corticosteroid insufficiency in critically ill adult patients: Consensus statements from an international task force by the American College of Critical Care Medicine. Crit. Care Med..

[42] Annane D., Pastores S.M., Rochwerg B., Arlt W., Balk R.A., Beishuizen A., Briegel J., Carcillo J., Christ-Crain M., Cooper M.S. (2017). Guidelines for the diagnosis and management of Critical Illness-Related Corticosteroid Insufficiency (CIRCI) in critically ill patients (Part I): Society of Critical Care Medicine (SCCM) and European Society of Intensive Care Medicine (ESICM) 2017. Crit. Care Med..

[43] Waterhouse R. (1911). A case of suprarenal apoplexy. Lancet.

[44] Friderichsen C. (1918). Nebennierenapoplexie bei kleinen Kindern. Jahrb. Kinderheilkd..

[45] Sharshar T., Annane D., de la Grandmaison G.L., Brouland J.P., Hopkinson N.S., Gray F. (2004). The Neuropathology of Septic Shock. Brain Pathol..

[46] McCann S.M., Kimura M., Karanth S., Yu W.H., Mastronardi C.A., Rettori V. (2006). The Mechanism of Action of Cytokines to Control the Release of Hypothalamic and Pituitary Hormones in Infection. Ann. N. Y. Acad. Sci..

[47] Polito A., Sonneville R., Guidoux C., Barrett L., Viltart O., Mattot V., Siami S., de la Grandmaison G.L., Chrétien F., Singer M. (2011). Changes in CRH and ACTH Synthesis during Experimental and Human Septic Shock. PLoS ONE.

[48] Bornstein S.R. (2009). Predisposing Factors for Adrenal Insufficiency. N. Engl. J. Med..

[49] Boonen E., van den Berghe G. (2014). Endocrine responses to critical illness: Novel insights and therapeutic implications. J. Clin. Endocrinol. Metab..

[50] Polito A., de la Grandmaison G.L., Mansart A., Louiset E., Lefebvre H., Sharshar T., Annane D. (2010). Human and experimental septic shock are characterized by depletion of lipid droplets in the adrenals. Intensive Care Med..

[51] Yen P.M. (2001). Physiological and molecular basis of Thyroid hormone action. Physiol. Rev..

[52] Michalaki M., Vagenakis A.G., Makri M., Kalfarentzos F., Kyriazopoulou V. (2001). Dissociation of the Early Decline in Serum T 3 Concentration and Serum IL-6 Rise and TNFα in Nonthyroidal Illness Syndrome Induced by Abdominal Surgery. J. Clin. Endocrinol. Metab..

[53] Peeters R.P., Wouters P.J., Kaptein E., van Toor H., Visser T.J., van den Berghe G. (2003). Reduced activation and increased inactivation of thyroid hormone in tissues of critically ill patients. J. Clin. Endocrinol. Metab..

[54] Yildizdaş D., Onenli-Mungan N., Yapicioğlu H., Topaloğlu A.K., Sertdemir Y., Yüksel B. (2004). Thyroid hormone levels and their relationship to survival in children with bacterial sepsis and septic shock. J. Pediatr. Endocrinol. Metab..

[55] Rodriguez-Perez A., Palos-Paz F., Kaptein E., Visser T.J., Dominguez-Gerpe L., Alvarez-Escudero J., Lado-Abeal J. (2008). Identification of molecular mechanisms related to nonthyroidal illness syndrome in skeletal muscle and adipose tissue from patients with septic shock. Clin. Endocrinol..

[56] Yanni G.N., Destariani C.P., Lubis A.N., Deliana M. (2019). Thyroid hormone profile in children with sepsis: Does euthyroid sick syndrome exist?. Open Access Maced. J. Med. Sci..

[57] Lin X., Shi S., Shi S. (2016). Sepsis leads to thyroid impairment and dysfunction in rat model. Tissue Cell.

[58] Lee S., Farwell A.P. (2016). Euthyroid Sick Syndrome. Comprehensive Physiology.

[59] Selvaraj N., Bobby Z., Sridhar M.G. (2008). Is euthyroid sick syndrome a defensive mechanism against oxidative stress?. Med. Hypotheses.

[60] Qin S.L., He Q., Hu L., He C.Y., Gao L.C., Young C.A., Chen J., Jiang C.F., Luo X.F., Zhou Y. (2018). The relationship between inflammatory factors, oxidative stress and DIO-1 concentration in patients with chronic renal failure accompanied with or without euthyroid sick syndrome. J. Int. Med. Res..

[61] van den Berghe G. (2016). On the neuroendocrinopathy of critical illness: Perspectives for feeding and novel treatments. Am. J. Respir. Crit. Care Med..

[62] Langouche L., Vander Perre S., Marques M., Boelen A., Wouters P.J., Casaer M.P., Van den Berghe G. (2013). Impact of early nutrient restriction during critical illness on the nonthyroidal illness syndrome and its relation with outcome: A randomized, controlled clinical study. J. Clin. Endocrinol. Metab..

[63] Mebis L., Eerdekens A., Güiza F., Princen L., Derde S., Vanwijngaerden Y.M., Vanhorebeek I., Darras V.M., Van den Berghe G., Langouche L. (2012). Contribution of nutritional deficit to the pathogenesis of the nonthyroidal illness syndrome in critical illness: A rabbit model study. Endocrinology.

[64] Lim C.F., Docter R.O., Visser T.J., Krenning E.P., Bernard B., Van Toor H., De Jong M., Hennemann G. (1993). Inhibition of thyroxine transport into cultured rat hepatocytes by serum of nonuremic critically ill patients: Effects of bilirubin and nonesterified fatty acids. J. Clin. Endocrinol. Metab..

[65] den Brinker M., Joosten K.F., Visser T.J., Hop W.C., de Rijke Y.B., Hazelzet J.A., Boonstra V.H., Hokken-Koelega A.C. (2005). Euthyroid sick syndrome in meningococcal sepsis: The impact of peripheral thyroid hormone metabolism and binding proteins. J. Clin. Endocrinol. Metab..

[66] Van der Poll T., Van Zee K.J., Endert E., Coyle S.M., Stiles D.M., Pribble J.P., Catalano M.A., Moldawer L.L., Lowry S.F. (1995). Interleukin-1 receptor blockade does not affect endotoxin-induced changes in plasma thyroid hormone and thyrotropin concentrations in man. J. Clin. Endocrinol. Metab..

[67] Fliers E., Guldenaar S.E.F., Wiersinga W.M., Swaab D.F. (1997). Decreased Hypothalamic Thyrotropin-Releasing Hormone Gene Expression in Patients with Nonthyroidal Illness 1. J. Clin. Endocrinol. Metab..

[68] Van den Berghe G., De Zegher F., Veldhuis J.D., Wouters P., Gouwy S., Stockman W., Weekers F., Schetz M., Lauwers P., Bouillon R. (1997). Thyrotrophin and prolactin release in prolonged critical illness: Dynamics of spontaneous secretion and effects of growth hormone-secretagogues. Clin. Endocrinol..

[69] Peeters R.P., Debaveye Y., Fliers E., Visser T.J. (2006). Changes within the thyroid axis during critical illness. Crit. Care Clin..

[70] van den Berghe G. (2014). Non-thyroidal illness in the ICU: A syndrome with different faces. Thyroid.

[71] Van den Berghe G., De Zegher F., Baxter R.C., Veldhuis J.D., Wouters P., Schetz M., Verwaest C., Van der Vorst E., Lauwers P., Bouillon R. (1998). Neuroendocrinology of Prolonged Critical Illness: Effects of Exogenous Thyrotropin-Releasing Hormone and Its Combination with Growth Hormone Secretagogues 1. J. Clin. Endocrinol. Metab..

[72] Angelousi A.G., Karageorgopoulos D.E., Kapaskelis A.M., Falagas M.E. (2011). Association between thyroid function tests at baseline and the outcome of patients with sepsis or septic shock: A systematic review. Eur. J. Endocrinol..

[73] Foks M., Dudek A., Polok K., Nowak-Kózka I., Fronczek J., Szczeklik W. (2019). Thyroid hormones as potential prognostic factors in sepsis. Anaesthesiol. Intensive Ther..

[74] Borkowski J., Siemiatkowski A., Wołczyński S., Czaban S.L., Jedynak M. (2005). Assessment of the release of thyroid hormones in septic shock—prognostic significance. Pol. Merkur. Lekarski.

[75] Kothiwale V.A., Patil P., Gaur S. (2018). Correlation of Thyroid Hormone Profile with the Acute Physiology and Chronic Health Evaluation II Score as a Prognostic Marker in Patients with Sepsis in the Intensive Care Unit. J. Assoc. Physicians India.

[76] Giustina A., Veldhuis J.D. (1998). Pathophysiology of the Neuroregulation of Growth Hormone Secretion in Experimental Animals and the Human 1. Endocr. Rev..

[77] Perchard R., Clayton P.E. (2017). Ghrelin and growth. Dev. Biol. Gastrointest. Horm..

[78] Arvat E., Maccario M., Di Vito L., Broglio F., Benso A., Gottero C., Papotti M., Muccioli G., Dieguez C., Casanueva F.F. (2001). Endocrine activities of ghrelin, a natural growth hormone secretagogue (GHS), in humans: Comparison and interactions with hexarelin, a nonnatural peptidyl GHS, and GH-releasing hormone. J. Clin. Endocrinol. Metab..

[79] Carreira M.C., Crujeiras A.B., Andrade S., Monteiro M.P., Casanueva F.F. (2013). Ghrelin as a GH-releasing factor. Endocr. Dev..

[80] Dahn M.S., Lange M.P., Jacobs L.A. (1988). Insulinlike Growth Factor 1 Production Is Inhibited in Human Sepsis. Arch. Surg..

[81] Dahn M.S., Lange M.P. (1998). Systemic and splanchnic metabolic response to exogenous human growth hormone. Surgery.

[82] Baxter R.C., Hawker F.H., To C., Stewart P.M., Holman S.R. (1998). Thirty-day monitoring of insulin-like growth factors and their binding proteins in intensive care unit patients. Growth Horm. IGF Res..

[83] Yumet G., Shumate M.L., Bryant P., Lin C.M., Lang C.H., Cooney R.N. (2002). Tumor necrosis factor mediates hepatic growth hormone resistance during sepsis. Am. J. Physiol. Endocrinol. Metab..

[84] Hong-Brown L.Q., Brown C.R., Cooney R.N., Frost R.A., Lang C.H. (2003). Sepsis-induced muscle growth hormone resistance occurs independently of STAT5 phosphorylation. Am. J. Physiol. Endocrinol. Metab..

[85] Langouche L., van den Berghe G. (2006). The Dynamic Neuroendocrine Response to Critical Illness. Endocrinol. Metab. Clin. N. Am..

[86] Adams J.M., Otero-Corchon V., Hammond G.L., Veldhuis J.D., Qi N., Low M.J. (2015). Somatostatin is essential for the sexual dimorphism of gh secretion, corticosteroid-binding globulin production, and corticosterone levels in mice. Endocrinology.

[87] Lang C.H., Pollard V., Fan J., Traber L.D., Traber D.L., Frost R.A., Gelato M.C., Prough D.S. (1997). Acute alterations in growth hormone-insulin-like growth factor axis in humans injected with endotoxin. Am. J. Physiol. Regul. Integr. Comp. Physiol..

[88] Baričević I., Jones D.R., Dordević B., Malenković V., Nedić O. (2007). Differential influence of open surgery and sepsis on the circulating insulin-like growth factors and their binding proteins as representative metabolic markers. Clin. Biochem..

[89] de Groof F., Joosten K.F., Janssen J.A., De Kleijn E.D., Hazelzet J.A., Hop W.C., Uitterlinden P., van Doorn J., Hokken-Koelega A.C. (2002). Acute stress response in children with meningococcal sepsis: Important differences in the growth hormone/insulin-like growth factor I axis between nonsurvivors and survivors. J. Clin. Endocrinol. Metab..

[90] Önenli-Mungan N., Yildizdas D., Yapicioglu H., Topaloglu A.K., Yüksel B., Özer G. (2004). Growth hormone and insulin-like growth factor 1 levels and their relation to survival in children with bacterial sepsis and septic shock. J. Paediatr. Child Health.

[91] Papastathi C., Mavrommatis A., Mentzelopoulos S., Konstandelou E., Alevizaki M., Zakynthinos S. (2013). Insulin-like Growth Factor I and its binding protein 3 in sepsis. Growth Horm. IGF Res..

[92] van den Berghe G. (2002). Dynamic neuroendocrine responses to critical illness. Front. Neuroendocrinol..

[93] Gu Y., Wang C., Cohen A. (2004). Effect of IGF-1 on the balance between autophagy of dysfunctional mitochondria and apoptosis. FEBS Lett..

[94] Li Y., Shelat H., Geng Y.J. (2012). IGF-1 prevents oxidative stress induced-apoptosis in induced pluripotent stem cells which is mediated by microRNA-1. Biochem. Biophys. Res. Commun..

[95] Xu L., Zhang W., Sun R., Liu J., Hong J., Li Q., Hu B., Gong F. (2017). IGF‑1 may predict the severity and outcome of patients with sepsis and be associated with microRNA‑1 level changes. Exp. Ther. Med..

[96] Kojima M., Hosoda H., Date Y., Nakazato M., Matsuo H., Kangawa K. (1999). Ghrelin is a growth-hormone-releasing acylated peptide from stomach. Nature.

[97] Chang L., Zhao J., Yang J., Zhang Z., Du J., Tang C. (2003). Therapeutic effects of ghrelin on endotoxic shock in rats. Eur. J. Pharmacol..

[98] Maruna P., Gürlich R., Frasko R., Rosicka M. (2005). Ghrelin and leptin elevation in postoperative intra-abdominal sepsis. Eur. Surg. Res..

[99] Waseem T., Duxbury M., Ito H., Ashley S.W., Robinson M.K. (2008). Exogenous ghrelin modulates release of pro-inflammatory and anti-inflammatory cytokines in LPS-stimulated macrophages through distinct signaling pathways. Surgery.

[100] Wang P. (2010). Mechanism of the inhibitory effect of ghrelin in sepsis. Hepatic Med. Evid. Res..

[101] Wan S.X., Shi B., Lou X.L., Liu J.Q., Ma G.G., Liang D.Y., Ma S. (2016). Ghrelin protects small intestinal epithelium against sepsis-induced injury by enhancing the autophagy of intestinal epithelial cells. Biomed. Pharmacother..

[102] Zhou M., Yang W.L., Aziz M., Ma G., Wang P. (2017). Therapeutic effect of human ghrelin and growth hormone: Attenuation of immunosuppression in septic aged rats. Biochim. Biophys. Acta Mol. Basis Dis..

[103] Spratt D.I. (2001). Altered gonadal steroidogenesis in critical illness: Is treatment with anabolic steroids indicated?. Best Pract. Res. Clin. Endocrinol. Metab..

[104] Samson W.K., Taylor M.M., Baker J.R. (2003). Prolactin-releasing peptides. Regul. Pept..

[105] Savino W. (2017). Prolactin: An Immunomodulator in Health and Disease. Front. Horm. Res..

[106] Guo H., Calkins J.H., Sigel M.M., Lin T. (1990). Interleukin-2 is a potent inhibitor of Leydig cell steroidogenesis. Endocrinology.

[107] Dong Q., Hawker F., McWilliam D., Bangah M., Burger H., Handelsman D.J. (1992). Circulating immunoreactive inhibin and testosterone levels in men with critical illness. Clin. Endocrinol..

[108] Wang C., Chan V., Yeung R.T. (1978). Effect of surgical stress on pituitary-testicular function. Clin. Endocrinol..

[109] Wang C., Chan V., Tse T.F., Yeung R.T.T. (1978). Effect of acute myocardial infarction on pituitary-testicular function. Clin. Endocrinol..

[110] Sharshar T., Bastuji-Garin S., De Jonghe B., Stevens R.D., Polito A., Maxime V., Rodriguez P., Cerf C., Outin H., Touraine P. (2010). Hormonal status and ICU-acquired paresis in critically ill patients. Intensive Care Med..

[111] Christeff N., Benassayag C., Carli-Vielle C., Carli A., Nunez E.A. (1988). Elevated oestrogen and reduced testosterone levels in the serum of male septic shock patients. J. Steroid Biochem..

[112] Woolf P.D., Hamill R.W., McDonald J.V., Lee L.A., Kelly M. (1985). Transient hypogonadotropic hypogonadism caused by critical illness. J. Clin. Endocrinol. Metab..

[113] Vanhorebeek I., Langouche L., den van den Berghe G. (2006). Endocrine aspects of acute and prolonged critical illness. Nat. Clin. Pract. Endocrinol. Metab..

[114] Berghe G., Zegher F., Lauwers P., Veldhuls J.D. (1994). Luteinizing hormone secretion and hypoandrogenaemia in critically ill men: Effect of dopamine. Clin. Endocrinol..

[115] Van den Berghe G., Weekers F., Baxter R.C., Wouters P., Iranmanesh A., Bouillon R., Veldhuis J.D. (2001). Five-Day Pulsatile Gonadotropin-Releasing Hormone Administration Unveils Combined Hypothalamic-Pituitary-Gonadal Defects Underlying Profound Hypoandrogenism in Men with Prolonged Critical Illness 1. J. Clin. Endocrinol. Metab..

[116] Mechanick J.I., Nierman D.M. (2006). Gonadal steroids in critical illness. Crit. Care Clin..

[117] Breen K.M., Karsch F.J. (2004). Does Cortisol Inhibit Pulsatile Luteinizing Hormone Secretion at the Hypothalamic or Pituitary Level?. Endocrinology.

[118] Breen K.M., Stackpole C.A., Clarke I.J., Pytiak A.V., Tilbrook A.J., Wagenmaker E.R., Young E.A., Karsch F.J. (2004). Does the type II glucocorticoid receptor mediate cortisol-induced suppression in pituitary responsiveness to gonadotropin-releasing hormone?. Endocrinology.

[119] Kalantaridou S.N., Makrigiannakis A., E Zoumakis G.P. (2004). Chrousos, Stress and the female reproductive system. J. Reprod. Immunol..

[120] Vardas K., Apostolou K., Briassouli E., Goukos D., Psarra K., Botoula E., Tsagarakis S., Magira E., Routsi C., Nanas S. (2014). Early Response Roles for Prolactin Cortisol and Circulating and Cellular Levels of Heat Shock Proteins 72 and 90α in Severe Sepsis and SIRS. BioMed Res. Int..

[121] Moss M. (2005). Epidemiology of Sepsis: Race, Sex, and Chronic Alcohol Abuse. Clin. Infect. Dis..

[122] Sakr Y., Elia C., Mascia L., Barberis B., Cardellino S., Livigni S., Fiore G., Filippini C., Ranieri V.M. (2013). The influence of gender on the epidemiology of and outcome from severe sepsis. Crit. Care.

[123] Fleischmann C., Thomas-Rueddel D.O., Hartmann M., Hartog C.S., Welte T., Heublein S., Dennler U., Reinhart K. (2016). Fallzahlen und sterblichkeitsraten von sepsis-patienten im krankenhaus. Dtsch. Arztebl. Int..

[124] Nasir N., Jamil B., Siddiqui S., Talat N., Khan F.A., Hussain R. (2015). Mortality in sepsis and its relationship with gender. Pakistan, J. Med. Sci..

[125] Xu J., Tong L., Yao J., Guo Z., Lui K.Y., Hu X., Cao L., Zhu Y., Huang F., Guan X. (2019). Association of Sex with Clinical Outcome in Critically Ill Sepsis Patients: A Retrospective Analysis of the Large Clinical Database MIMIC-III. Shock.

[126] Sunden-Cullberg J., Nilsson A., Inghammar M. (2020). Sex-based differences in ED management of critically ill patients with sepsis: A nationwide cohort study. Intensive Care Med..

[127] de Farias L.M., de Brito D., Sales J.P., Rodrigues R.S., de Meneses F.A. (2011). Gender and mortality in sepsis: Do sex hormones impact the outcome?. Rev. Bras. Ter. Intensiva.

[128] El-Lakany M.A., Fouda M.A., El-Gowelli H.M., El-Gowilly S.M., El-Mas M.M. (2018). Gonadal hormone receptors underlie the resistance of female rats to inflammatory and cardiovascular complications of endotoxemia. Eur. J. Pharmacol..

[129] Saia R.S., Garcia F.M., Cárnio E.C. (2015). Estradiol protects female rats against sepsis induced by Enterococcus faecalis improving leukocyte bactericidal activity. Steroids.

[130] Zeng Z., Surewaard B.G., Wong C.H., Guettler C., Petri B., Burkhard R., Wyss M., Le Moual H., Devinney R., Thompson G.C. (2018). Sex-hormone-driven innate antibodies protect females and infants against EPEC infection. Nat. Immunol..

[131] Vázquez-Martínez E.R., García-Gómez E., Camacho-Arroyo I., González-Pedrajo B. (2018). Sexual dimorphism in bacterial infections. Biol. Sex Differ..

[132] Norbury W.B., Jeschke M.G., Herndon D.N. (2007). Metabolism modulators in sepsis: Propranolol. Crit. Care Med..

[133] Lang C.H. (1992). Sepsis-induced insulin resistance in rats is mediated by a β-adrenergic mechanism. Am. J. Physiol. Endocrinol. Metab..

[134] Deibert D.C., DeFronzo R.A. (1980). Epinephrine-induced insulin resistance in man. J. Clin. Investig..

[135] Andersen S.K., Gjedsted J., Christiansen C., Tønnesen E. (2004). The roles of insulin and hyperglycemia in sepsis pathogenesis. J. Leukoc. Biol..

[136] Lönnroth P., Smith U. (1983). β-Adrenergic dependent downregulation of insulin binding in rat adipocytes. Biochem. Biophys. Res. Commun..

[137] Chu C.A., Sindelar D.K., Igawa K., Sherck S., Neal D.W., Emshwiller M., Cherrington A.D. (2000). The direct effects of catecholamines on hepatic glucose production occur via α1- and β2-receptors in the dog. Am. J. Physiol. Endocrinol. Metab..

[138] Randle P.J., Garland P.B., Hales C.N., Newsholme E.A. (1963). The glucose fatty-acid cycle. Its role in insulin sensitivity and the metabolic disturbances of diabetes mellitus. Lancet.

